# When Early Experiences Build a Wall to Others’ Emotions: An Electrophysiological and Autonomic Study

**DOI:** 10.1371/journal.pone.0061004

**Published:** 2013-04-10

**Authors:** Martina Ardizzi, Francesca Martini, Maria Alessandra Umiltà, Mariateresa Sestito, Roberto Ravera, Vittorio Gallese

**Affiliations:** 1 Dept. of Neuroscience, Section of Physiology, University of Parma, Parma, Italy; 2 IIT (Italian Institute of Technology) Brain Center for Social and Motor Cognition, Parma, Italy; 3 Dept. of Psychology, ASL 1 (Azienda Sanitaria Locale) Imperiese, Imperia, Italy; University of South Florida, United States of America

## Abstract

Facial expression of emotions is a powerful vehicle for communicating information about others’ emotional states and it normally induces facial mimicry in the observers. The aim of this study was to investigate if early aversive experiences could interfere with emotion recognition, facial mimicry, and with the autonomic regulation of social behaviors. We conducted a facial emotion recognition task in a group of “street-boys” and in an age-matched control group. We recorded facial electromyography (EMG), a marker of facial mimicry, and respiratory sinus arrhythmia (RSA), an index of the recruitment of autonomic system promoting social behaviors and predisposition, in response to the observation of facial expressions of emotions. Results showed an over-attribution of anger, and reduced EMG responses during the observation of both positive and negative expressions only among street-boys. Street-boys also showed lower RSA after observation of facial expressions and ineffective RSA suppression during presentation of non-threatening expressions. Our findings suggest that early aversive experiences alter not only emotion recognition but also facial mimicry of emotions. These deficits affect the autonomic regulation of social behaviors inducing lower social predisposition after the visualization of facial expressions and an ineffective recruitment of defensive behavior in response to non-threatening expressions.

## Introduction

Early adverse experiences disrupt emotional development since they may exert negative impact on affect recognition, social engagement and self-regulation. These competencies have rarely been measured among a) youth living in extreme poverty and neglect and b) by using objective behavioral and physiological measures which are invulnerable to self-report. The goal of this paper is to examine deficits in social, affective and physiological regulation among children living in extreme adversity in post-conflict Sierra Leone.

The exposure to early adverse experiences results in a set of symptoms, described among children and adults [1], which exceeds the traditional diagnosis of Post Traumatic Stress Disorder (PTSD) [2]. The peculiarity of early aversive experiences is that they divert the resources normally dedicated to growth and development to support mere survival [3]. In this way they prevent the complete and healthy development of secure attachment relationships, of a stable and integrated self-concept and of competence to self-regulate emotions and behaviors [4]. These skills are normally fostered by a secure caregiving context [5], [6]. Consequently, exposure to early aversive experiences produces chronic and severe problems in affect recognition, interpersonal relationships and self-regulation [7]. The facial expression of emotions is a powerful vehicle of information by which humans communicate emotional states to each others. Its great communicative power enables the immediate understanding of others’ dispositions, which provides the basis for consistent social behavioral responses. An immediate acceleration of the heart rate in front of an angry face or a deceleration of pulse when we see others smiling commonly result. Behavioral studies showed that infant abuse [8]–[11], neglect [8], and trauma [12], all disrupt development of emotion recognition skills producing a perceptual bias for angry facial expressions to the exclusion of other negative emotions (e.g., sadness). On the basis of EEG studies [13], [14] these results have been interpreted as a specific form of learning from early social experiences. Children and young adults adapt their pre-existing perceptual and attentive mechanisms to process environmental aspects that become especially salient [11], [15]. Could early aversive experiences also affect facial mimicry in response to the observation of the facial expression of emotions and the related autonomic regulation of social behavior? People exposed to pictures of negative and positive emotional facial expressions, spontaneously [16] and rapidly [17] produce distinct facial electromyographic (EMG) reactions in the same muscles involved in expressing identical positive and negative emotions. This phenomenon, called “facial mimicry”, has been proposed to facilitate empathy, emotional reciprocity and recognition [18]–[22].

The Polyvagal Theory provides a conceptual framework for understanding the link between autonomic subsystems and the regulation of social behaviors [23]–[26]. The Polivagal Theory describes three anatomically and physiologically different autonomical subsystems each of which is respectively linked to immobilization (e.g., feigning death, promoted by unmyelinated vagus nerve), mobilization (e.g., fight–flight behaviors, fostered by sympathetic nervous system), and social communication (e.g., displaying facial expressions, making eye contact, expressing prosody, and modulating the middle ear muscles to improve the extraction of human voice, mediated by myelinated vagal fibers) [26]. The last subsystem, unique to mammals, rapidly regulates the cardiac output working as a ‘‘vagal brake’’. When the “vagal brake” is applied, heart rate decreases to promote calm behavioral states and foster social interaction. According to this perspective, the autonomic nervous system would work as part of a social engagement system [23]–[26] enabling individuals to assess risk and, if the environment is perceived as safe, to inhibit ineffective defensive behaviors and to recruit the subsystem associated with social communication. The dynamic influence of the ”vagal brake” can be monitored by quantifying the amplitude of respiratory sinus arrhythmia (RSA; [27]). Lower baseline RSA indicates the removal of the “vagal brake” that may potentiate defensive behaviors (e.g., fight/flight or immobilization) and interfere with the ability to regulate behavior to socially engage with others. In contrast, higher baseline RSA suggests the activation of the “vagal brake” to promote social predisposition, emotion expression and self-regulation skills [24]. Recently, besides baseline RSA, attention has been given to RSA suppression. Higher baseline and greater RSA suppression are considered indexes of better ability to engage and disengage with the demands of environmental requests [28]. Accordingly, individuals with higher baseline RSA should show greater RSA suppression to meet metabolic demands of taxing environmental conditions, including threatening stimuli.

The aim of this study was to investigate if early aversive experiences could affect facial mimicry in response to facial displays of emotion and the related autonomic regulation of social behaviors. A link between spontaneous social behaviors (e.g. facial expressions) and autonomic bodily states becomes possible due to the neuroanatomical and neurophysiological integration between the nuclei that regulate the muscles of the face and the myelinated vagus [26]. Greater facial expressivity, in particular in the upper face, is associated with higher level of RSA [25]. On the other hand, a lower facial mimicry could be associated with defensive behavior (e.g., fight/flight or immobilization), instead of social engagement. An example and extreme case of aversive early experience is the condition of street-boys in Sierra Leone who live alone on the street without any type of parental/social care. We defined “street-boys” according to the Report of the National Head Count of Street Children in Sierra Leone as “any child whose life is controlled by the streets. […] Any child who spends the majority of his or her time on the street, regardless of whether he or she has a home to return to at night [29]. The impact of this condition is amplified when the juvenile population is the largest and there are lots of children living in the street. In Sierra Leone 49,698 street-boys are identified [29] and 41.7% of the population of the country is under 15 years old [30].

A forced-choice facial emotion recognition task was conducted on a group of street-boys and on a control group of age-matched boys, both from Sierra Leone. In agreement with previous results [8]–[10], [12] we expected to find a bias for angry faces among street-boys but not among controls. If a bias for anger were present this label should be over-attributed to other negative emotions. Facial EMG and RSA during the observation of facial expressions of emotions were also recorded. The aversive environment in which street-boys grow-up is characterized by pronounced competition as opposed to collaboration since resources are quite limited. In this context an empathic resonance to others’ emotions, by means of facial mimicry, is not adaptive. Accordingly, we expected to find lower mimicry response to facial expressions in street-boys but not in controls. A deficit of facial mimicry, developed as an adaptation to aversive early experiences, could diminish social understanding and magnify defensive behavior. If this were the case, lower baseline RSA after stimuli visualization and non-functional RSA suppression during the visualization of non-threat facial expressions should be expected among street-boys.

## Method

### Participants

A total of 60 participants were recruited for the study. Participants with cardio-respiratory or psychiatric diseases, users of drugs interfering with the cardio-respiratory activity and heavy smokers (>25 cigarettes per day; [31]) were excluded. Moreover, participants who had more than 30% of trials rejected for artifacts were also excluded. These exclusion criteria resulted in a final sample of 41 participants. Of these 19 were street-boys recruited in the Juvenile Prison of Freetown [Street-Boys group (SBg); mean age 15.7 years SE 0.3; mean years of schooling 7.1 SE 0.8; all males] and 22 were students in the Holy Family School of Freetown [Control group (Cg); mean age 15.9 years SE 0.3; mean years of schooling 5.3 SE 0.7; all males].

We conducted the Boston Naming Test (BNT; [32]) to exclude any differences between groups in labeling skill and to avoid a confounding effect on behavioral results. No differences were found between groups in BNT performance [SBg: 24.3 SE 1.8; Cg: 22.7 SE 0.9; t_39_ = 0.87, p = 0.39], age [t_39_ = −0.44, p = 0.66], and years of schooling [t_33_ = 1.52, p = 0.14]. Most participants came from the ethnic group of Krio and Temne, 34% and 32% of the sample, respectively. All participants in the SBg were street-boys in their first-time in jail; 73% of SBg had committed and experienced intense physical violence. They were all characterized by an extreme level of social deprivation, marginalization and neglect in the years preceding their arrest. Most had left parents’ home before the age of ten and on average they lived in the street for six years without the presence of a stable adult figure in their lives. None of the participants in the Cg were street-boys or had ever been in prison. They lived with their parents or close relatives. Written informed consent was obtained from the legal tutors and guardians of participants. Verbal consent was obtained from participants. A local social worker explained to the participants how the research would be conducted and asked if they would be available to collaborate with us. We sought oral consent from the participants, with the social worker serving as witness since, some of them were illiterate and most better understood the oral instruction given by the social worker. The experimental protocol, and the modalities by which the informed consent was collected were approved by the Ethics Committee of ASL1 of Imperia, Italy and by the Ethics Committee of the Ministry of Health and Sanitation of the Republic of Sierra Leone.

### Stimuli

Stimuli were 64 video-morphing constructed by using the Montreal Facial Displays of Emotion stimulus set (MSFDE; [33]). The video-morphing, lasting 3000 msec (15 fps; 800×560 pixels), showed the transition from a neutral facial expression to an emotional one (16 anger, 16 fear, 16 joy and 16 sadness). Each emotion expression was modeled by Asian, African, Hispanic and Caucasian actors balanced for gender (4 stimuli for each ethnic group, 2 males and 2 females). Stimuli were presented using E-Prime 2.0 software (Psychology Software Tools, Inc).

### Procedure

The experimental session consisted of two experiments: the behavioral experiment was a forced-choice facial emotion recognition task and the physiological experiment was a simple perceptive task conducted to record EMG and RSA responses to the stimuli. The behavioral and physiological responses were recorded in two separate experimental sessions to avoid potentially confounding effects of verbal responses and of mental-stress occurring during emotion recognition task on electrophysiological measurements [34]–[36]. The order of the experiments was balanced between participants. The entire experimental session for both groups of participants was conducted using the same experimental setting and at the same location, the seat of the Murialdo’s Josephites Mission in Kissy (Freetown). All participants of the SBg obtained a special permit to leave the Juvenile Prison for the time of the experiments. A local social assistant was always present to ensure that participants remained at ease, understood the instructions and to translate from English to Krio, if necessary.

#### Behavioral experiment

Participants sat comfortably at a table, in front of a monitor (1024X768@75Hz). They were asked to pay attention and to observe each stimulus for its entire duration. Stimuli were presented once (64 trials, 16 trials for each emotion: anger, fear, joy and sadness) in random order. After each stimulus, with no time limit, participants were asked to identify which of the four alternative labels (anger, fear, joy, sadness) best described the emotion expressed in the stimulus. The four alternatives were always visible and written in English and Krio on a sheet of paper. Participants’ answers were verbally expressed and transcribed by the experimenter. Before each stimulus presentation the question “you able du am?” (i.e. “Are you ready?”) appeared on the monitor, after the participant’s affirmative answer the experimenter pressed the spacebar to show the successive stimulus. The behavioral experiment lasted approximately 15 min.

#### Physiological experiment

Participants were required to abstain from alcohol, caffeine and tobacco for 2 hours prior to the experiment (see [37]). They sat comfortably at a table in front of a monitor (1024X768@75Hz). Participants were asked to carefully observe the videos. The physiological experiment consisted of four “condition-blocks” (each lasting 192 sec) and two “baseline-blocks” (each lasting 120 sec). The four “condition-blocks”, one for each emotional condition (anger, fear, joy and sadness), were randomly presented. Inside each “condition-block” the sixteen stimuli, comprising the same emotion, were randomly presented three times (48 trials). Each stimulus was preceded by a fixation cross lasting one second. The two “baseline-blocks”, consisting of a black centered fixation cross on gray background, were performed one before (Baseline) and one after (Recovery) the four “condition-blocks”. Overall the physiological experiment lasted 17 min. Electrophysiological responses were recorded for its entire duration. In order to maintain participants’ attention, after each “condition-block” the experimenter posed a question about the videos just shown. Participants’ faces were video-recorded to ensure that they looked at the screen.

### Facial EMG Data Acquisition and Reduction

Facial EMG activity was bipolarly recorded on the left side of the face with 4 mm standard Ag/Ag-Cl electrodes. Before being attached over the Corrugator Supercilii and the Zygomaticus Major muscle regions [38] the electrodes were filled with gel electrode paste and the participants’ skin was cleaned with an alcohol solution. Data were converted and amplified with an eight-channel amplifier (PowerLab8/30; ADInstruments UK) and displayed, stored, and reduced with LabChart 7.3.1 software package (ADInstruments, 2011). Facial EMG were sampled at 2 kHz and recorded with an online Mains Filter (adaptive 50 Hz filter). A 20–500 Hz band-pass filter [39] was applied offline on the raw facial EMG signal. The average amplitude of the EMG signal was obtained with the root-mean-square method [38]. Following standard practice [40], [41], EMG response (expressed in microvolts) was measured as change scores representing the difference between activity during each 500 msec of the 3 sec stimulus period and the 500 msec of the fixation cross immediately preceding stimulus onset. EMG signal and video recordings were visually inspected off line by the experimenters. In order to remove artifacts, EMG data that coincided with participants’ contingent movements (e.g. coughing, talking or whole head movements) were excluded from the analysis. Moreover, trials with mean change scores that were 2 SD above or below the grand mean change score calculated for each participant were considered outliers and removed.

### ECG Data Acquisition and Reduction

Three Ag/AgCl pre-gelled electrodes (ADInstruments, UK) with a contact area of 10 mm diameter were placed in an Einthoven's triangle configuration to monitor ECG (Powerlab and OctalBioAmp8/30, ADInstruments, UK). The ECG was sampled at 1 kHz and online filtered with the Mains Filter. The peak of the R-wave of the ECG was detected from each sequential heartbeat. The R-R intervals were extracted and the artifacts edited by integer division or summation [42]. Editing consisted of visual detection of outlier points, typically caused by failure to detect an R-peak (e.g., edit via division) or faulty detections of two or more ‘‘peaks’’ within a period representing the R-R interval (e.g., edit via summation) [42]. The amplitude of RSA was quantified with CMetX (available from http://apsychoserver.psych.arizona.edu) that produces data with a correlation near the unity with those obtained using Boher & Porges method [43]. The amplitude of RSA [expressed in ln(msec)^2^] was calculated as the variance of heart rate activity across the band of frequencies associated with spontaneous respiration (0.12–0.40 Hz) [43]. RSA was extracted for the first 2 min of each “condition-block” and each “baseline-block”, according to guidelines [42]. Mean Baseline value was the mean of Baseline and Recovery RSA values. The RSA suppression value for each “condition block” was measured as a change of scores between the RSA of the “condition block” and the one of Mean Baseline.

## Results

### Behavioral Results

**Figure 1:**
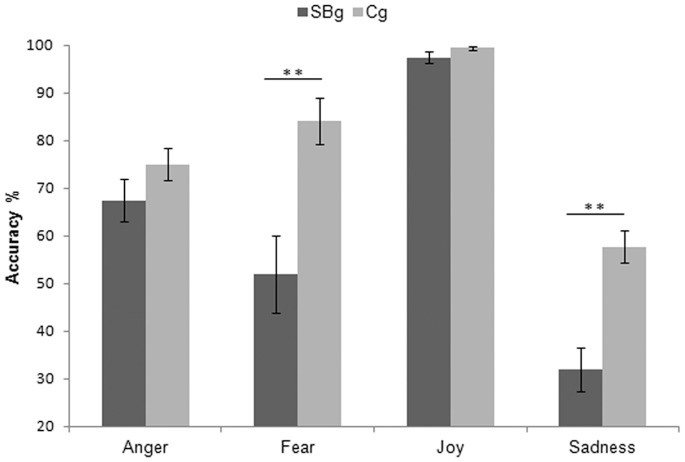
Accuracy Rate. Accuracy rate for Street-Boys group (SBg) and Control group (Cg). ** = p<0.005. Only differences between groups were showed. See the text for differences inside each group. Error bars are SE.

A repeated-measures ANOVA was performed on accuracy rate (% of correct response) in forced-choice facial emotion recognition task with Group (SBg, Cg) as between-factor and Emotion (Anger, Fear, Joy, Sadness) as within-factor. Results showed that the factor Group was significant (F_1,39_ = 27.15 p<0.005) as was the factor Emotion (F_3,117_ = 55.35 p<0.005), as well as the interaction of Emotion by Group (F_3,117_ = 5.91 p<0.005). Post hoc analyses, all conducted using LSD test, revealed that SBg had lower accuracy rate than Cg (SBg: 62.17% SE 3.76; Cg: 79.05% SE 2.32, p<0.005), and that Joy was the most recognized emotion (98.48% SE 0.61) while Sadness was the less recognized one (45.73% SE 3.44). Joy and Sadness were, indeed, significantly different from all other emotions (all p_s_<0.005). Post hoc analysis on the interaction Emotion by Group showed that the two groups differed in their accuracy rates to Fear (SBg: 51.97% SE 8.17, Cg: 84.09% SE 4.79) and Sadness (SBg: 31.91% SE 4.58, Cg: 57.67% SE 3.44) (all p_s_<0.005). Within the SBg accuracy rates for each emotion differed from each other (all p_s_<0.05), being Joy (97.37% SE 1.20) the most recognized emotion followed by Anger (67.43% SE 4.37), Fear (51.97% SE 8.17) and Sadness (31.91% SE 4.58). Within the Cg, Joy (99.43% SE 0.39) was the most recognized emotion followed by Fear (84.09% SE 4.79), Anger (75.00% SE 3.32) and Sadness (57.67% SE 3.34); the accuracy rates for Anger and Fear were not significantly different (p>0.05) (Figure 1).

**Figure 2:**
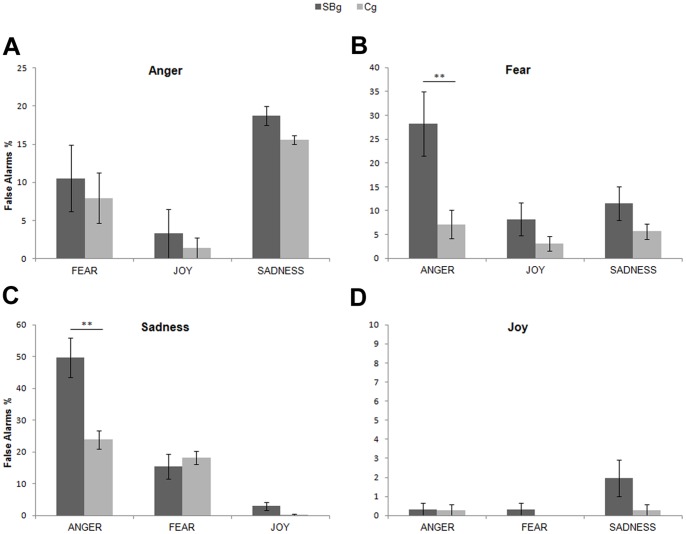
False Alarms. False Alarms rate displayed condition by condition for Street-Boys group (SBg) and Control group (Cg). ** = p<0.005. Only differences between groups were showed. See the text for differences inside each group. Error bars are SE.

We evaluated the presence of the bias conducting an analysis of False-Alarms (FA the emotion label selected by participants did not match the stimulus’s emotion). Four separate repeated-measures ANOVAs were performed on FA rate (% of incorrect use of each label calculated inside each condition) with Group (SBg, Cg) as between-factor and FA (3 levels) as within-factor. For the Anger condition the two groups did not differ (F_1,39_ = 1.96 p>0.05), but there was a main effect of FA (F_2,78_ = 23.18 p<0.005). The post hoc test on FA showed that the mistaken use of the three labels differed significantly from each other: sadness was the most used one (17.07% SE 1.99), followed by fear (9.15% SE 1.65) and joy (2.29% SE 0.64) (all p_s_<0.005). Interaction FA by Group was not significant (F_2,78_ = 0.04 p>0.05) (Figure 2 panel A).

For the Fear condition, the factor Group was significant (F_1,39_ = 12.27 p<0.005), as the factor FA (F_2,78_ = 6.56 p<0.005) and the interaction FA by Group (F_2,78_ = 3.44 p<0.05). The post hoc analysis on the two main effects revealed that SBg committed significantly more FA than the Cg (SBg: 16% SE 2.97; Cg: 5.30% SE 1.24; p<0.005), and that anger was the most attributed label, being significantly different from all others (16.92% SE 3.84; all p_s_<0.05). The post hoc test of the interaction FA by Group revealed that the two groups differed only for the use of the anger label (SBg: 28.29% SE 6.73; Cg: 7.10% SE 3.00; p<0.005). Moreover, within the SBg anger differed from all other labels (all p_s_<0.005), whereas inside the Cg labels were all used equally often (all p_s_>0.40) (Figure 2 panel B).

For the Sadness condition, the factor Group was significant (F_1,39_ = 20.87 p<0.005), as the factor FA (F_2,78_ = 40.22 p<0.005) and the interaction FA by Group (F_2,78_ = 8.91 p<0.005). The post hoc on the main effect of Group revealed that SBg committed significantly more FA than the Cg (SBg: 22.70% SE 3.59; Cg: 14.10% SE 1.71; p<0.005). The post hoc on the main effect FA revealed that all labels differed among each other: anger was the most attributed one (35.82% SE 3.79), followed by fear (16.92% SE 2.12) and joy (1.52% SE 0.64) (all p_s_<0.005). The post hoc on the interaction FA by Group showed that SBg attributed more often the label anger than the Cg (SBg: 49.67% SE 6.17; Cg: 23.86% SE 2.87; p<0.005). Within the SBg the wrong use of each label was different from the others (anger: 49.67% SE 6.17; fear: 15.46% SE 3.89; joy: 2.96% SE 1.30; all p_s_<0.05). On the other hand, inside the Cg the incorrect attribution of joy differed from all other labels (0.28% SE 0.28 all p_s_<0.05) being the less used one, while no differences were found between anger and fear (p>0.24) (Figure 2 panel C).

Finally, the same analysis conducted on Joy condition showed that neither the factor Group (F_1,39_ = 3.00 p>0.05), nor the factor FA (F_2,78_ = 3.11 p>0.05), nor the interaction FA by Group (F_2,78_ = 2.22 p>0.05) were significant (Figure 2 panel D).

### Physiological Results

A repeated measures ANOVA was performed on facial EMG responses with Group (SBg, Cg) as between-factor and with Muscle (Corrugator, Zygomaticus), Emotion (Anger, Fear, Joy, Sadness) and Epoch (six epochs lasting 500 msec) as within-factors.

The analysis revealed that the main effects of Group (F_1,39_ = 24.84 p<0.005), Muscle (F_1,39_ = 9.10 p<0.05) and Emotion (F_3,117_ = 6.30 p<0.05) were significant as were the interactions Muscle by Emotion (F_3,117_ = 22.00 p<0.005), Muscle by Emotion by Group (F_3,117_ = 5.75 p<0.005), Emotion by Epoch (F_15,585_ = 2.32 p<0.005) and the interaction Muscle by Emotion by Epoch (F_15,585_ = 5.40 p<0.005). The post hoc test on the main effects of Group and Muscle showed that the SBg (−0.46 µV SE 0.03) had lower EMG responses than the Cg (0.15 µV SE 0.03) (p<0.005), and that the Corrugator muscle (−0.01 µV SE 0.02) was more activated than the Zygomaticus (−0.25 µV SE 0.03) (p<0.05). The post hoc on the main effect of Emotion revealed that Joy (0.10 µV SE 0.05) differed from all other emotions, showing the highest EMG responses (all p_s_<0.05). The post hoc on the interaction Muscle by Emotion showed that the Corrugator responses during each emotion condition were significantly different from the Zygomaticus ones (all p_s_<0.005), moreover within each muscle Joy EMG response was different from all other activations, being the highest for the Zygomaticus (0.45 µV SE 0.09; all p_s_<0.005) and the lowest for the Corrugator (−0.24 µV SE 0.02, all p_s_<0.05).

**Figure 3:**
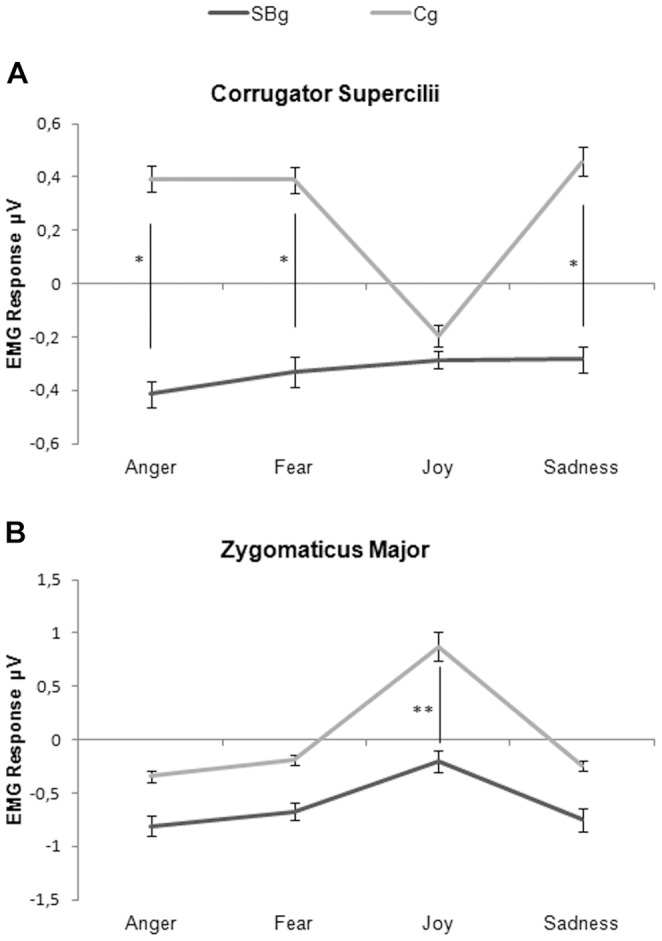
EMG responses. Corrugator Supercilii (A) and Zygomaticus Major (B) EMG responses for Street-Boys group (SBg) and Control group (Cg) during presentation of emotional facial expressions. * = p<0.05; ** = p<0.005. Only differences between groups were showed. See the text for differences inside each group. Error bars are SE.

Importantly, the post hoc conducted on the interaction Muscle by Emotion by Group showed that the Corrugator responses of the SBg during Anger, Fear and Sadness conditions were significantly lower than the respective Corrugator responses of the Cg (all p_s_<0.05). Within the SBg the Corrugator responses during all emotions conditions were not significantly different (all p_s_>0.42); otherwise, within the Cg the response during Joy condition was significantly different from the other conditions (all p_s_<0.005) (Figure 3 panel A). For the Zygomaticus, the response during Joy condition significantly differed between the two groups, being SBg responses lower than Cg (p<0.005). In both groups responses during Joy condition were significantly different from all other conditions (SBg all p_s_<0.05; Cg all p_s_<0.005) (Figure 3 panel B). The post hoc of the interaction Emotion by Epoch wasn’t discussed because less informative than the following interaction lacking the significant factor Muscle. The post hoc of the interaction Muscle by Emotion by Epoch revealed that the two muscles activations, inside each emotion condition, were significantly different during the entire duration of the stimuli (all p_s_<0.005). The Corrugator muscle did not show any significant difference among epochs for any emotion (all p_s_>0.06). The lack of modulation among different epochs was also found for the Zygomaticus muscle for all negative emotions (Anger, Fear, Sadness), but not for Joy. In this condition Zygomaticus EMG signal in each epoch was different from all others excluding the firsts two (all p_s_<0.05), with increasing EMG signal in relation with the intensity of the observed emotion.

**Figure 4:**
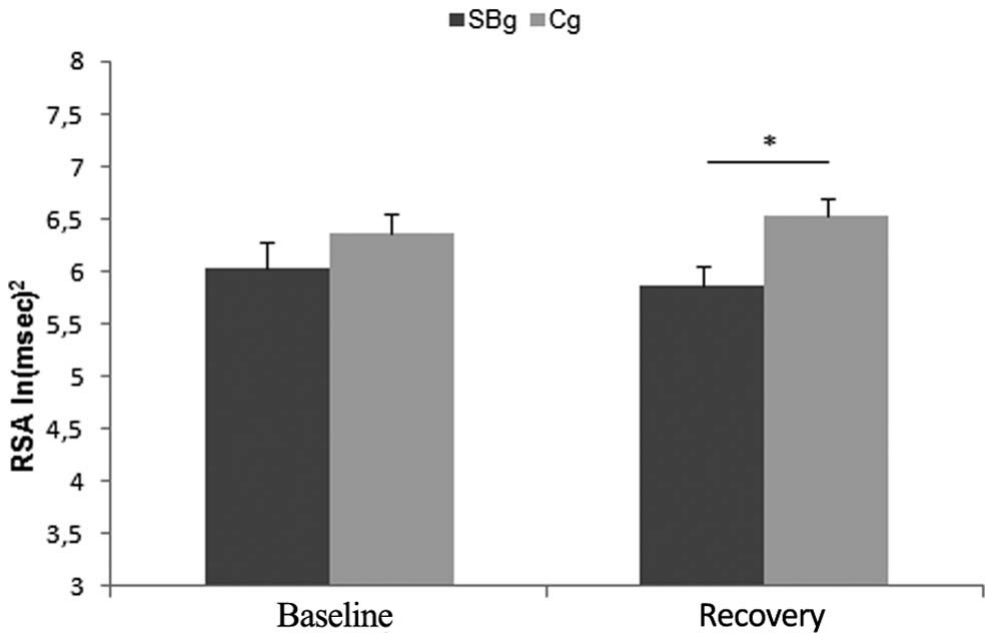
Baseline RSA values. Baseline and Recovery RSA values for Street-Boys group (SBg) and Control group (Cg). * = p<0.012. Error bars are SE.

Finally, we conducted four different independent sample T-tests with a Bonferroni-corrected significance level of p<.012 (calculated as alpha level/number of observations = 0.05/4) on Baseline and Recovery RSA values. Five participants (two from the SBg and three from the Cg) were excluded from this analysis being outliers (±2 SD), thus the analysis on autonomic data concerned 17 street-boys and 19 controls. First, we evaluated the presence of significant differences between the two groups, in Baseline and Recovery RSA values. A significant difference was found only for Recovery (Baseline: T_34_ = −1.07, p>0.05; Recovery: T_34_ = −2.72, p<0.012) (Figure 4). The SBg (5.86 ln(msec)^2^ 0.21) showed a lower RSA value during Recovery than Cg (6.53 ln(msec)^2^ SE 0.16). Second, it is well known that during situations demanding sustained attention, RSA is suppressed [44]. To disentangle this possible confounding effect on our results, we contrasted Baseline and Recovery of each group. If an attentional effect were present, Recovery should be significantly lower than Baseline. No significant difference was found between Baseline and Recovery within SBg (Baseline: 6.04 ln(msec)^2^ SE 0.25; Recovery: 5.86 ln(msec)^2^ SE 0.19; T_16_ = 1.31, p>0.05) and Cg (Baseline: 6.36 ln(msec)^2^ SE 0.18; Recovery: 6.53 ln(msec)^2^ SE 0.16; T_19_ = −1.59, p>0.05) (Figure 4).

**Figure 5:**
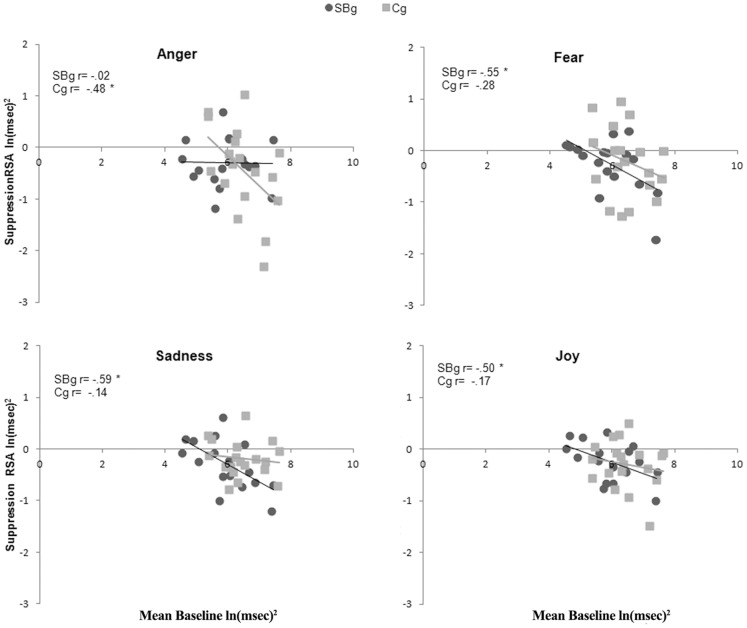
Correlation between Mean Baseline and Suppression RSA. Correlation between Mean Baseline and Suppression RSA values for Street-Boys group (SBg) and Control group (Cg) inside each condition. * = p<0.05.

In order to verify the existence of a significant correlation between Mean Baseline RSA and the levels of RSA suppression measured during the presentation of threatening stimuli, we performed two correlation analyses (one for each group) between the Mean Baseline value and the RSA suppression values of each condition (see [45]). Interestingly, SBg showed significant negative correlation in Fear (r_17_ = −0.55, p<0.05), Sadness (r_17_ = −0.59, p<0.05) and Joy (r_17_ = −0.50, p<0.05) but not in Anger (r_17_ = −0.02, p>0.05) conditions. Otherwise, as expected, we found a significant negative correlation in Anger condition only, for the Cg (r_19_ = −0.49, p<0.05) (Figure 5). In other words, among SBg higher Mean baseline RSA corresponded to higher RSA suppression during the visualization of Fear, Sadness and Joy facial expressions. Among Cg the same correlation was found only during Anger presentation.

## Discussion

The goal of this study was to investigate if early aversive experiences could affect facial emotion recognition, facial mimicry, and the related autonomic regulation of social behavior. We evaluated, at a behavioral level, the presence among street-boys of the well known bias for anger [8]–[10], [12]. SBg showed less accuracy than Cg in sadness and fear conditions, moreover the analysis of FA revealed that SBg erroneously used the anger label significantly more often than other labels and than Cg, exclusively in these conditions. In agreement with previous studies, our results confirmed that SBg participants, but not controls, showed a biased emotion recognition favoring anger, to the detriment of recognition of fear and sadness. Given the ethno-cultural homogeneity of the two groups of participants, we can exclude that the significant difference between the two groups might be due to cultural bias.

At the physiological level the electrophysiological and autonomic responses to facial expressions of the SBg, manifested severe impairments. Corrugator responses during negative emotions and Zygomaticus responses during positive emotion of street-boys were significantly weaker than those of controls. Furthermore, the SBg’s Corrugator responses did not show any modulation for pleasant and unpleasant stimuli [38], [40], [46]. Lower Corrugator EMG response during negative emotion was already found in women reporting high-betrayal abuse in childhood [47]. Differing from our results, Reichmann-Decker et al. [47] found stronger Zygomaticus responses during positive emotions with respect to control group. These authors interpreted this result as an attempt to cope with an hostile environment. It is possible that age and gender might influence strategies adopted to cope with an adverse environment. Further research will be required to shed light on this point.

Lack of difference in Corrugator responses during observation of positive and negative emotions was described in ASD children [48], who typically showed deficit in empathic resonance [49]. To the best of our knowledge no previous study reported a similar effect in children or young adults with a past history of abuse or maltreatment. The unspecific activation of the Corrugator in SBg reported here cannot be due to attentional effects. As concluded by previous studies, abused children show a behavioral bias to anger emotional stimuli caused by an alteration of attentional processing [13], [14]. Granted this behavioral bias also among street-boys, if attention modulated the EMG activation, stronger Corrugator EMG responses to angry faces should have been found. We actually found the opposite.

Taken together, the present results suggest that early aversive experiences affect processes supporting empathy, emotional reciprocity and emotion recognition [21], [22]. The effects of early aversive experiences were not restricted to electrophysiological EMG responses. At the autonomic level there was a significant higher Recovery RSA value for the Cg than for the SBg. These results suggest that the observation of emotional facial expressions induces higher social predisposition [24] in the Cg than in the SBg. The effects of early aversive experiences on autonomic regulation were investigated among female adolescents exposed to child maltreatment, who exhibited lower baseline RSA than controls [50], and among adults showing a deficit in rapidly re-engaging vagal regulation immediately after mild exercise [51]. Thus, early experiences affect the abilities to recruit autonomic regulation levels promoting social communication, not simply at a baseline level [50], or after a non-social task [51], but also after exposure to social stimuli like the facial expression of emotions, as our data clearly demonstrate.

The failure of SBg to recruit autonomic regulation levels promoting social communication is coherent with the modulation of RSA during the visualization of facial expression of emotions. As expected, controls showed a significant correlation between Mean Baseline and RSA suppression during anger condition, suggesting an efficient release of the “vagal brake” [24], [28] in response to potentially threatening stimuli. On the contrary, street-boys showed a significant correlation between Mean Baseline and RSA suppression for non-threatening conditions (fear, sadness and joy) but not during the observation of angry faces. This suggests that street-boys interpreted fear, sadness and joy as potentially threatening stimuli. This in turn led to the recruitment of the autonomic subsystem promoting defensive behaviors (fight/flight or immobilization), instead of the one enabling social communication and predisposition. The nature of our data cannot clarify which was the exact type of defensive strategies adopted by street-boys. They could suppress RSA to promote a sympathetic reaction (fight/flight) or a vagal response (immobilization). The lack of facial mimicry seems to be more consistent with the recruitment of a freezing response to facial expression of emotions. Indeed it was shown that people exposed to multiple life aversive events exhibited a freezing response to pleasant and unpleasant affective pictures [52], suggesting that aversive life events could promote generalized freezing response to social stimuli instead of prosocial and empathic behavioral responses. The absence of an effective suppression of the “vagal brake” during the observation of faces expressing anger could be likely due to a relatively low intensity of angry stimuli for individuals normally accustomed to more severe levels of anger expression as suggested by Pollak et al. [53] It is possible that in order to perceive threat in angry faces and to rapidly predispose defensive responses, street-boys required more intense facial expressions of anger.

The present data demonstrate that street-boys not only showed impairment of facial emotion recognition and facial mimicry, but also of the autonomic regulation of social behaviors.

### Limitations

The SBg could not be included in a single category of maltreatment (e.g. physical abuse, neglect, trauma), as done in previous studies. Street-boys lived in pervasive and perennial aversive conditions: they experienced physical violence from peers and adults, were abandoned by their family and, at the moment of the experiment, they were serving a prison sentence. For this reason we were not able to evaluate the different impact of single experiences on our sample. Questionnaires evaluating cognitive and psychological aspects were not administered due to the lack of validated scales on this kind of sample. Finally, since only males were included in the experiment, our conclusions may not extend to females.

### Conclusion

Our study demonstrates that pervasive aversive early experiences not only affect facial emotion recognition but also the correlated EMG physiological responses and the autonomic regulation of social behaviors. The environment in which street-boys lived likely promoted the suppression of emotional mimicry, building a wall between their and others’ emotions. Alteration of empathy, emotional reciprocity and emotion recognition, normally fostered by processes like facial mimicry, also affected the autonomic regulation of social behaviors, inducing lower social predisposition after the visualization of facial expressions and an ineffective recruitment of defensive behavior in response to non-threatening expressions. These conclusions acquire further importance if we consider that accurate recognition and response to social emotional signals, like facial expressions, facilitate adaptive social functioning. Difficulties in affect recognition, lower facial expressivity, blunted and dysregulated RSA response are common characteristics also among clinical samples like children with autism [48], [54] and antisocial personality disorder [55]–[57]. Failure of these processes can promote the development of antisocial behaviors and ineffective coping strategies. Such negative effects are amplified in a social context such as the one studied here, where high numbers of young boys live in conditions of extreme marginalization and maltreatment. Understanding the processes through which early social experiences affect child development increases the likelihood of effective prevention and intervention programs. Our findings confirm that in the case of exposure to early aversive experiences it would be necessary, besides the traditional attention to PTSD symptoms [7], also the development of treatments focused on affect recognition, self regulation and social engagement. Moreover, being these deficits associated with physiological alterations and not only with cognitive or explicit processes [11], [15], interventions should appeal to these physiological aspects to induce recovery of affective and social skills.

Further research is needed to determine if treatments promoting facial mimicry to emotional expressions could ameliorate emotion recognition and the correlated autonomic regulation of social behaviors.
